# Supplementary material for: Efficacy of defocus incorporated multiple segments (DIMS) lenses and low-dose atropine on retarding myopic shift among premyopic preschoolers: Protocol for a prospective, multicenter, randomized controlled trial
Source: PLoS One. 2024 Dec 31;19(12):e0312935. doi: 10.1371/journal.pone.0312935 (PMC11687691; doi:10.1371/journal.pone.0312935)
Supplement: S2 File — (DOC) [file pone.0312935.s002.doc]

**國立陽明交通大學附設醫院人體試驗委員會**

**計畫書中文摘要**

- 1. 計畫名稱： [兒童近視前期的早期介入計畫](https://ymuh.cims.tw/wiPtms/protocolStatus.do?protocolId=350)
  2. 研究主持人、研究人員及試驗進行地點：

1. 研究主持人：楊欣瑜醫師
2. 研究人員：蔡德中醫師、王喬瑜醫師、楊雨潔醫師
3. 試驗進行地點：國立陽明交通大學附設醫院
   1. 研究計畫經費來源：國科會
   2. 研究背景：兒童近視已成為全球性的問題，尤其在亞洲國家，近視普及率與高度近視的比率逐年增加，根據研究，屆2050年約有一半以上的人口會有近視的問題。近視除了造成生活上的不方便，也會造成可觀的、甚至致盲的眼科疾病例如白內障、青光眼、黃斑病變與視網膜剝離。如何防治幼兒近視的發生與近視進展是目前各國政府在努力的方向。現常用控制近視的方法除了鼓勵戶外活動、減少近距離與螢幕使用，另外也有醫療的方法介入，例如睫狀肌麻痺劑亞托平眼藥水、單焦近視眼鏡、周邊離焦近視控制眼鏡、角膜塑型片及日拋離焦隱形眼鏡。現階段的醫療主要在治療與控制已經近視的族群，而在近視前期(pre-myopic status)的兒童則無一致的預防與治療共識。
   3. 研究目的：研究周邊離焦近視控制眼鏡在近視前期兒童使用之影響。
   4. 研究型態：□侵入性介入研究 █非侵入性介入研究 □病歷回溯研究

(可複選) █問卷調查研究 □研究用人體檢體採集 □剩餘檢體

□質性研究 □流行病學 □既有資料分析

□其它，請說明：＿＿＿＿＿＿＿

- 1. 研究設計：
  2. 進行方式：平行研究設計
  3. 對照組為相同為近視前期而無使用任何治療之兒童
  4. 盲化方式：非盲
  5. 隨機分派：隨機分派，依照病患與家屬同意後進行分組
  6. 研究方法：收集近視前期兒童分別使用離焦眼鏡、亞托平眼藥水與觀察組相互對照，了解使用離焦眼鏡之安全性，藉由眼科醫師檢查追蹤兒童眼睛健康與近視之變化，問券調查兒童近視相關風險、使用不同預防方法的方便性與滿意度等。
  7. 健保資料庫研究█不適用；□適用，請加填本項

1. 本申請案之初始目的為(可複選):

□ 申請使用健保資料庫(準備向衛生福利部申請資料現場分析)

□ 申請研究計畫

□ 已有健保資料，擬投稿發表

□ 已有健保資料，擬用於學位論文研究

□ 其他

2. 研究方法：

□ case-control study

□ cohort study: prospective, retrospective

□ cross-sectional study

□ Nested case-control study

□ case-cohort study

□ other:

- 1. 檢體研究█不適用；□適用，請加填本項

1. 檢體採集方法：
2. 採集種類：□血液，數量 (c.c)；□體液： ，數量 (c.c)

□其他 ，數量(大小)

1. 所採集之檢體或其衍生物是否進行生物醫學研究(指與基因等生物基本特徵有關之醫學研究)：□是；□否
2. 檢體或其衍生物是否提供、讓與或授權國立陽明交通大學附設醫院以外之他人使用：

□否

□是，可能□提供□讓與□授權 (□國內，機構名稱： ；

□國外，機構名稱： )

1. 檢體是否與其他資料庫或病歷資料連結：

□否

□是，資料庫名稱 ，隸屬於 機構或單位

病歷資料內容：□病歷號碼 □姓名 □性別□出生日期 □入出院日期

□診斷名稱 □手術名稱 □其他：

1. 檢體資料處理方式(單選)

□資料仍保有可供辨識個人之資訊 □以編碼識別 □加密 □完全去連結

7. 計畫結束後剩餘檢體之處理方式：

□銷毀 □保存，保存地點 保存多久

- 1. 受試者隱私保護與資訊安全管理：

1. 資料收集方式(可複選)：

█病歷資料 █問卷 □訪談 □攝影或照像 □其他，請說明

2. 資料處理方式(單選)

□資料仍保有可供辨識個人之資訊 █以編碼識別 □加密 □完全去連結

*** 編碼**：指以代碼取代參與者姓名、國民身分證統一編號、病歷號等可供辨識之個人資訊，使達到難以辨識個人身分之作業方式。

*** 加密**：指將足以辨識參與者個人身分之資料、訊息，轉化為無可辨識之過程。

*** 去連結**：指於生物檢體、資料、資訊編碼後，使其與可供辨識參與者之個人資料、資訊，永久無法以任何方式連結、比對之作業。

3. 可能接觸受試者資料之人員(可複選)：

█計畫主持人 █共(協)同主持人 □研究助理 □計畫監測稽核人員

□其他，請說明

4. 受試者資料保存方式：

█書面資料會上鎖保存，電腦資料設定密碼管控，僅限研究團隊成員使用。

□非以上述方式保存，請說明

5. 計畫結束後資料處理方式

□銷毀 █保存，保存地點 國立陽明交通大學附設醫院眼科門診 保存多久10年

- 1. 統計分析：

1. 描述統計：藉由問券調查，了解近視前期兒童使用離焦近視控制眼鏡之副作用與相關不適。比較使用離焦近視控制眼鏡與無使用治療之近視前期兒童，在追蹤後的近視控制情況。比較使用離焦近視控制眼鏡與使用亞托平眼藥水治療之近視前期兒童，在追蹤後的近視控制情況。
2. 推論統計：連續性的數據使用T test，而質性數據使用chi square檢定。
3. 期間分析：兒童分別在配戴離焦近視控制眼鏡後一週、一個月、三個月、半年、一年及二年填寫問券，做眼科檢查。
   1. 預期成果及主要效益：了解近視前期兒童配戴離焦近視控制眼鏡的感受與控制近視的效果。
   2. 研究成果之歸屬及運用：院內計畫及未來近視控制政策參考。研究成果歸屬於參與之研究人員。
   3. 研究人員利益衝突事項之揭露：

1.計畫主持人（申請人）或其關係人與計畫委託廠商或其負責人間近三年不曾有僱傭、委任或代理關係。2. 計畫主持人（申請人）或其關係人與計畫委託廠商或其負責人間近三年不曾有價格、利率等不符市場正常合理交易之資金借貸、投資、背書、保證等財務往來。3.計畫主持人（申請人）與計畫委託廠商負責人非為配偶或三親等以內之血親或姻親。4.計畫主持人（申請人）或其關係人非計畫委託廠商之董事、監察人或經理人。但以官股代表身分擔任董事或監察人者，不在此限。前項所稱之關係人，包含計畫主持人（申請人）之配偶、共同生活之家屬、二親等以內親屬及計畫主持人（申請人）或其配偶信託財產之受託人。

**Summary of Project Proposal**

**National Yang-Ming Chiao Tung University Hospital Human Research Ethics Committee**

**Project Title:** Early intervention for Premyopic children

**Principal Investigator, Researchers, and Study Location:**

- **Principal Investigator:** Dr. Hsin-Yu Yang
- **Researchers:** Dr. Der-Chong Tsai, Dr. Chiao-Yu Wang, Dr. Yu-Chieh Yang
- **Study Location:** National Yang-Ming Chiao Tung University Hospital
- **Funding Source:** National Science and Technology Council

**Research Background:** Myopia in children has become a global issue, particularly in Asian countries, where the prevalence and rate of high myopia are increasing annually. According to research, by 2050, more than half of the population will have myopia. Myopia not only causes inconvenience in daily life but also leads to significant and potentially blinding ophthalmic diseases such as cataracts, glaucoma, macular degeneration, and retinal detachment. Preventing the onset and progression of myopia in children is a focus of governmental efforts worldwide. Common current methods for controlling myopia include encouraging outdoor activities, reducing near work and screen time, and medical interventions like atropine eye drops, single-focus myopic glasses, peripheral defocus myopia control glasses, orthokeratology lenses, and daily disposable defocus contact lenses. The current medical approach primarily focuses on treating and controlling existing myopia, while there is no consensus on the prevention and treatment of children in the pre-myopic status.

**Research Objective:** To study the impact of peripheral defocus myopia control glasses on pre-myopic children.

**Research Type:**

- **Non-invasive Intervention Research**
- **Questionnaire Survey Research**

**Research Design:**

- **Methodology:** Parallel study design
- **Control Group:** Children with pre-myopia who do not receive any treatment
- **Blinding Method:** Non-blind
- **Random Assignment:** Randomly assigned with consent from patients and their families

**Research Method:** Collect data on pre-myopic children using defocus glasses, atropine eye drops, and the observation group. Assess the safety of defocus glasses through ophthalmic examinations, monitor changes in children's eye health and myopia, and conduct surveys on myopia-related risks, convenience, and satisfaction with different preventive methods.

**Protection of Participant Privacy and Information Security Management:**

1. **Data Collection Methods:**
   - Medical records
   - Questionnaires
2. **Data Processing Methods:**
   - Data will be coded for identification
3. **Possible Personnel Accessing Participant Data:**
   - Principal Investigator
   - Co-investigators
4. **Data Preservation Methods:**
   - Written data will be stored securely, and computer data will be password protected, accessible only to the research team.
5. **Data Handling After Project Completion:**
   - Data will be stored at the Ophthalmology Department of National Yang-Ming Chiao Tung University Hospital for 10 years.

**Statistical Analysis:**

- **Descriptive Statistics:** Use questionnaire surveys to understand the side effects and discomfort of pre-myopic children using defocus myopia control glasses. Compare the myopia control status of pre-myopic children using defocus myopia control glasses, those using atropine eye drops, and those without treatment after follow-up.
- **Inferential Statistics:** Use T-tests for continuous data and chi-square tests for qualitative data.
- **Periodic Analysis:** Children will fill out questionnaires and undergo ophthalmic examinations one week, one month, three months, six months, one year, and two years after wearing defocus myopia control glasses.

**Expected Outcomes and Main Benefits:** Understand the experiences and effectiveness of pre-myopic children wearing defocus myopia control glasses in controlling myopia.

**Attribution and Utilization of Research Results:** The results will belong to all participating researchers and will be used for internal projects and future myopia control policies.

**Disclosure of Conflicts of Interest for Researchers:**

1. The Principal Investigator or related persons have not had employment, commission, or agency relationships with the project commissioning manufacturer or its responsible person in the past three years.
2. There have been no financial transactions such as loans, investments, endorsements, or guarantees between the Principal Investigator or related persons and the project commissioning manufacturer or its responsible person that are not in line with market norms in the past three years.
3. The Principal Investigator and the project commissioning manufacturer’s responsible person are not spouses or relatives within three degrees of kinship.
4. The Principal Investigator or related persons are not directors, supervisors, or managers of the project commissioning manufacturer, except for directors or supervisors in the capacity of state-owned share representatives.

中文計劃書

1. 計畫名稱：[兒童近視前期的早期介入計畫](https://ymuh.cims.tw/wiPtms/protocolStatus.do?protocolId=350)
2. 研究主持人、研究人員及試驗進行地點：
3. 研究主持人：楊欣瑜
4. 研究人員：蔡德中醫師、王喬瑜醫師、楊雨潔醫師
5. 試驗進行地點：國立陽明交通大學附設醫院
6. 主持人研究機構：臺北榮民總醫院蘇澳暨員山分院
7. 研究計畫經費來源：國科會計畫
8. 研究背景：兒童近視已成為全球性的問題，尤其在亞洲國家，近視普及率與高度近視的比率逐年增加，根據研究，屆2050年約有一半以上的人口會有近視的問題。近視除了造成生活上的不方便，也會造成可觀的、甚至致盲的眼科疾病例如白內障、青光眼、黃斑病變與視網膜剝離。如何防治幼兒近視的發生與近視進展是目前各國政府在努力的方向。現常用控制近視的方法除了鼓勵戶外活動、減少近距離與螢幕使用，另外也有醫療的方法介入，例如睫狀肌麻痺劑亞托平眼藥水、單焦近視眼鏡、周邊離焦近視控制眼鏡、角膜塑型片及日拋離焦隱形眼鏡。現階段的醫療主要在治療與控制已經近視的族群，而在近視前期(pre-myopic status)的兒童則無一致的預防與治療共識。
9. 研究目的：研究周邊離焦近視控制眼鏡或低濃度睫狀肌麻痺劑亞托平在近視前期兒童使用之影響。
10. 研究對象及實施方法：招募360位近視前期兒童，隨機分派，分為對照組、使用低濃度雅托平與使用離焦鏡片眼鏡組。追蹤三組後續眼睛健康與近 視屈光度變化。
11. 計畫摘要：研究對照組、使用低濃度雅托平與使用離焦鏡片眼鏡組。追蹤三組兩年眼睛健康與近視屈光度變化。
12. 統計分析：
13. 描述統計：藉由問券調查，了解近視前期兒童使用離焦近視控制眼鏡或低濃度睫狀肌麻痺劑亞托平之影響。比較使用離焦近視控制眼鏡、使用睫狀肌麻痺劑亞托平和無使用治療之近視前期兒童，在追蹤後的近視控制情況。
14. 推論統計：連續性的數據使用T test，而質性數據使用chi square檢定。
15. 期間分析：兒童分別在一個月、三個月、半年、一年、一年三個月、一年六個月、一年九個月及二年填寫問券，做眼科檢查。
16. 計畫預定進度：113 年 2 月底完成收案，113 年 6 月底完成初步報告，114 年 6 月 完成初步統計，115 年 10 月發表成果。
17. 研究對象權益之保障、同意之方式：告知後同意。
18. 研究相關設備需求：國立陽明交通大學附設醫院眼科診間之設備。
19. 研究經費需求：擬申請國立陽明交通大學附設醫院院內計畫。
20. 研究人員利益衝突事項之揭露：無。
21. 預期成果及主要效益：了解近視前期兒童配戴離焦近視控制眼鏡的感受與控制近視 的效果。
22. 研究成果之歸屬及運用：院內計畫及未來近視控制政策參考。研究成果歸屬於所有 參與研究者。

**Project Proposal: Early Intervention for Premyopic children**

**Principal Investigator, Researchers, and Study Location:**

- **Principal Investigator:** Hsin-Yu Yang
- **Researchers:** Dr. Der-Chong Tsai, Dr. Chiao-Yu Wang, Dr. Yu-Chieh Yang
- **Study Location:** National Yang-Ming Chiao Tung University Hospital
- **Principal Investigator's Affiliated Institution:** Taipei Veterans General Hospital, Su-Ao and Yuanshan Branch
- **Funding Source:** National Science and Technology Council

**Research Background:** Myopia in children has become a global issue, especially in Asian countries where the prevalence and rate of high myopia are increasing yearly. According to research, by 2050, more than half of the population will have myopia. Myopia not only causes inconvenience in daily life but also leads to significant and potentially blinding ophthalmic diseases such as cataracts, glaucoma, macular degeneration, and retinal detachment. Preventing the onset and progression of myopia in children is a focus of governmental efforts worldwide. Current common methods for controlling myopia include encouraging outdoor activities, reducing near work and screen time, and medical interventions like atropine eye drops, single-focus myopic glasses, peripheral defocus myopia control glasses, orthokeratology lenses, and daily disposable defocus contact lenses. The current medical approach primarily focuses on treating and controlling existing myopia, while there is no consensus on the prevention and treatment of children in the pre-myopic status.

**Research Objective:** To study the impact of peripheral defocus myopia control glasses or low-concentration atropine on pre-myopic children.

**Study Subjects and Implementation Methods:** Recruit 360 pre-myopic children, randomly assigned to control groups, low-concentration atropine groups, and defocus lens groups. Follow up on the eye health and myopic refractive changes in these three groups.

**Project Summary:** Study the control group, low-concentration atropine group, and defocus lens group. Follow up on the eye health and myopic refractive changes in these three groups over two years.

**Statistical Analysis:**

- **Descriptive Statistics:** Use questionnaire surveys to understand the impact of peripheral defocus myopia control glasses or low-concentration atropine on pre-myopic children. Compare the myopia control status of pre-myopic children using defocus myopia control glasses, atropine, and no treatment after follow-up.
- **Inferential Statistics:** Use T-tests for continuous data and chi-square tests for qualitative data.
- **Periodic Analysis:** Children will fill out questionnaires and undergo eye exams at one month, three months, six months, one year, one year and three months, one year and six months, one year and nine months, and two years.

**Projected Schedule:**

- Complete recruitment by the end of February 113.
- Complete the preliminary report by the end of June 113.
- Complete preliminary statistics by June 114.
- Publish results in October 115.

**Protection of Participants' Rights and Consent:** Informed consent will be obtained.

**Research-Related Equipment Requirements:** Equipment from the Ophthalmology Department of National Yang-Ming Chiao Tung University Hospital.

**Funding Requirements:** Plan to apply for an internal project at National Yang-Ming Chiao Tung University Hospital.

**Disclosure of Conflicts of Interest for Researchers:** None.

**Expected Outcomes and Main Benefits:** Understand the experiences and effectiveness of pre-myopic children wearing defocus myopia control glasses in controlling myopia.

**Attribution and Utilization of Research Results:** The results will belong to all participants and will be used for internal projects and future myopia control policies.
