# Supplementary material for: Efficacy of defocus incorporated multiple segments (DIMS) lenses and low-dose atropine on retarding myopic shift among premyopic preschoolers: Protocol for a prospective, multicenter, randomized controlled trial
Source: PLoS One. 2024 Dec 31;19(12):e0312935. doi: 10.1371/journal.pone.0312935 (PMC11687691; doi:10.1371/journal.pone.0312935)
Supplement: Attachment — Submitted filename: Response to Reviewers.docx [file pone.0312935.s003.docx]

**Response to Reviewers**

We appreciate the reviewers' valuable feedback and suggestions. Below are our responses and the corresponding revisions made in the manuscript:

1. **File Naming**: The file names have been updated according to the style templates.
2. **Role of Funders**: The role of the funders in the study has been detailed in the cover letter and at the end of the manuscript.
3. **Data Availability**: The data availability statement has been updated to indicate that the data will be available upon acceptance.
4. **Supporting Information Captions**: Captions for the supporting information files have been added at the end of the manuscript.
5. **References**: The references have been thoroughly checked and are in accordance with the guidelines.

**Response** **to** **Reviewers’** **Comments**

**Reviewer #1:** **In this study protocol, a three-arm randomized control trial is being proposed to evaluate the efficacy of optical and pharmacological interventions in preventing myopia among premyopic preschoolers. The primary outcome is the mean change in cycloplegic spherical equivalent (SE). Secondary outcomes include the cumulative percentage of incident myopia, the cumulative percentage of a fast myopic shift of SE, and the changes in mean axial length.**

1. **Line 180: If block randomization will be used, indicate the block size.
   Response**: Thank you for the suggestion. We have added the block size of 3 in the manuscript. Please refer to page 7, line 181.
2. **Line 345: State the statistical testing method which achieves 80% power.
   Response**: We use analysis of variance (ANOVA) to achieve 80% power. This has been included in the manuscript. Please refer to page 16, lines 358–359.
3. **Statistical analysis (lines 356-366:) Repeated measures ANOVAs, testing the interactions of time by treatment effect, would be superior to standard ANOVAs and independent t-tests due to repeated testing.**

**Response**: Thank you for this important suggestion. We have changed our analysis to repeated measures ANOVA to better account for the repeated testing. Please see page 16, lines 372–374.

4. **Identify the software that will be used for statistical analysis.**

**Response**: The software used for the statistical analysis is IBM SPSS Statistics 26 (IBM Corp., Armonk, N.Y., USA). This has been added to the manuscript on page 16, lines 374–375.

**Reviewer #2: Dear authors,
You presented a well designed protocol in order to assess the effect of DIMS and low dose atropine on myopic evolution. Overall, the sections of the protocol were clearly explained, along with the endpoints and the statistical methods you will employ. I have no specific complaints regarding the protocol, which appears detailed and complete. Good luck for your trial.**

**Response**: Thank you for your positive and encouraging feedback on our study protocol. We are grateful for your acknowledgment of the study design and clarity in the protocol. We will continue to ensure rigorous implementation and follow-up throughout the trial. We look forward to sharing the results in due course.
